# Meteorological and Environmental Factors Associated With Sudden Cardiac Arrest During Marathons in Japan

**DOI:** 10.1016/j.jacadv.2026.102965

**Published:** 2026-06-29

**Authors:** Jo Kato, Tomohiro Manabe, Masao Iwagami, Fumihiro Yamasawa, Yasushi Honda

**Affiliations:** a Department of Cardiology, NHO Kasumigaura Medical Center, Tsuchiura, Japan; b Medical Committee, Japan Association of Athletics Federations (JAAF), Tokyo, Japan; c Sports Medicine Research Center, Keio University, Yokohama, Japan; d Department of Health Services Research, University of Tsukuba, Tsukuba, Japan; e Marubeni Health Promotion Center, Tokyo, Japan; f Faculty of Health and Sport Sciences, University of Tsukuba, Tsukuba, Japan

**Keywords:** air pollution, ambient temperature, marathon running, sudden cardiac arrest

## Abstract

**Background:**

Sudden cardiac arrest (SCA) during marathons is a rare but life-threatening event. Although underlying cardiovascular conditions have been implicated in race-related SCA, the contribution of environmental factors, including meteorological conditions and ambient air pollution, remains incompletely understood.

**Objectives:**

The objective of the study was to examine the associations of meteorological conditions and ambient air pollutants with SCA during marathon races.

**Methods:**

We conducted a nationwide race-level study in Japan using data from 4.53 million runners participating in Japan Association of Athletics Federations–certified full marathons between April 2011 and March 2020. Race-level SCA counts were linked to meteorological variables at race start, including ambient temperature, humidity, solar radiation, wind speed, precipitation, and air pressure, and to ambient air pollutants including fine particulate matter, suspended particulate matter, photochemical oxidants, sulfur dioxide, nitrogen dioxide, and carbon monoxide. Associations were examined using Poisson regression models with adjustment for race characteristics and participant composition.

**Results:**

Among 4.53 million runners, 75 cases of SCA were identified. Ambient temperature at race start was inversely associated with SCA risk (adjusted incidence rate ratio per 1 °C increase: 0.94; 95% CI: 0.90-0.99), corresponding to a higher risk at lower temperatures. No statistically significant associations were observed between ambient air pollutants and SCA risk.

**Conclusions:**

In Japanese marathon races held predominantly in autumn and winter, lower ambient temperature at race start was associated with a higher risk of SCA, whereas no significant association was observed for ambient air pollutants.

Sudden cardiac arrest (SCA) during sporting activities is a rare but devastating event. In road races, including marathons, SCA has been reported to occur at a rate of approximately 0.5 to 2 cases per 100,000 runners,^1^, ^2^, ^3^, ^4^ and remains a major concern in endurance sports. The Race Associated Cardiac Arrest Event Registry (RACER 2) study,^2^ which included more than 29 million participants in the United States, showed that nearly half of identified cases were attributable to coronary artery disease, consistent with findings from other national registries.^1^^,^^3^

Most previous studies of SCA during road races have focused on the characteristics of individual runners, whereas the contribution of environmental factors has received less attention. In the general population, meteorological conditions and air pollution have been associated with acute cardiovascular events, including cardiovascular morbidity and mortality.^5^, ^6^, ^7^ However, whether these environmental factors are associated with SCA during endurance running events remains insufficiently investigated.

Marathon races provide a distinctive epidemiological setting in which a large number of participants are exposed to broadly similar environmental conditions over a defined time period. Taking advantage of this setting, we linked a nationwide registry of SCA during Japan Association of Athletics Federations (JAAF)–certified marathon races with meteorological data from the Japan Meteorological Agency and air pollution data from the National Institute for Environmental Studies. Using this integrated data set, we investigated the associations of meteorological variables and ambient air pollutants with the risk of SCA during marathon races.

## Methods

### Study design and setting

This race-level observational study used a nationwide prospective registry of SCA during marathon races in Japan. The study was reported in accordance with the Strengthening the Reporting of Observational Studies in Epidemiology (STROBE) guidelines.^8^ The study was approved by the Institutional Ethics Committee of the Sports Medicine Research Center, Keio University (No. 2013-03), and the requirement for individual informed consent was waived because no personally identifiable information was collected.

### Study population and case ascertainment

**Figure 1:**
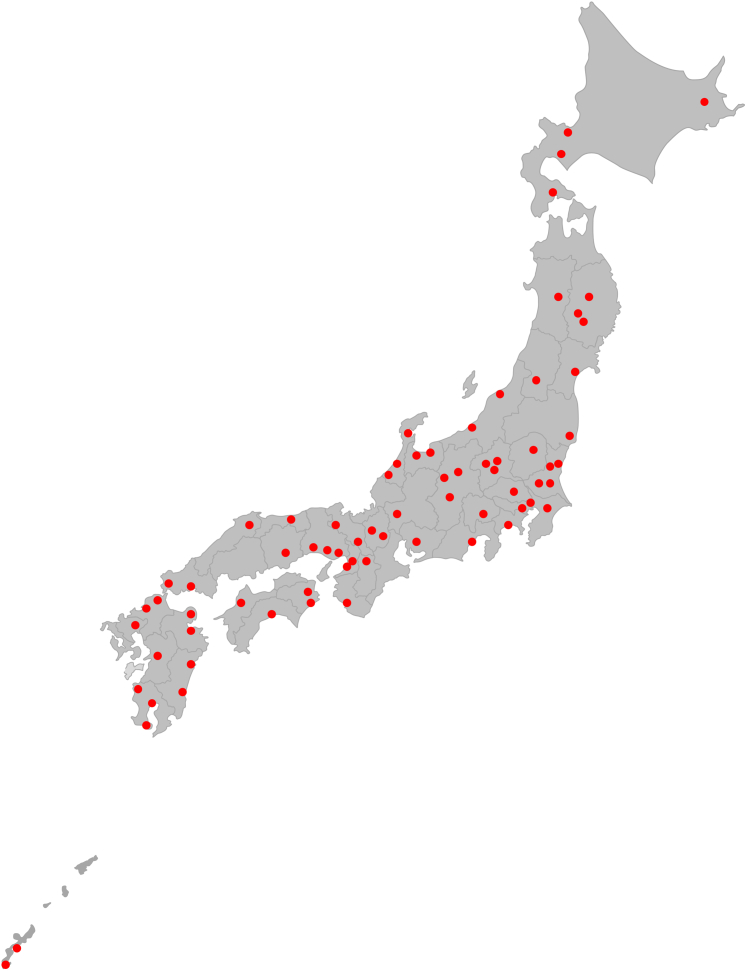
Distribution of Japan Association of Athletics Federations-Certified Marathon Races Red dots indicate the location of each marathon race. For races held in close geographical proximity, one of the locations is omitted for clarity.

The JAAF certifies more than 70 full marathon races (26.2 miles) annually across 42 of the 47 prefectures in Japan (Figure 1). Since April 2011, the JAAF Medical Committee has maintained a nationwide registry of SCA during marathon races.^9^ Members of the medical committee sent questionnaires to race offices shortly after each race regarding race-day medical events and medical response activities. Local newspaper reports and internet news articles were also reviewed to identify potentially unreported cases, and potential cases identified through these sources were confirmed by direct contact with race offices. SCA was defined operationally as a sudden collapse occurring between the race start and 1 hour after the finish that required resuscitative measures, including chest compressions and/or use of an automated external defibrillator, as ascertained by race medical personnel. All cases were subsequently transported to hospital irrespective of return of spontaneous circulation. To maximize questionnaire response rates and case ascertainment, the specific etiology of each event was not pursued.

The number of marathon participants was defined as the number of runners who crossed the start line. Age-group data were available for 290 races; for additional races, age composition was estimated from registrant or finisher lists,^9^ yielding usable age-group data for 460 races (80.6%). Participants were categorized as aged <40 years, 40 to 59 years, or ≥60 years. Sex distribution was based on self-reported registration information.

The route of JAAF-certified races follows World Athletics rules,^10^ which require the straight-line distance between the start and finish points to be <50% of the total course length. Race start was used as a common exposure window within each event, and environmental variables at that time were considered broadly representative of overall race-day conditions.

### Meteorological and air pollutant variables

Weather data were obtained from the Japan Meteorological Agency, which operates 56 meteorological observatories and approximately 1,300 automated meteorological data acquisition system stations across Japan.^11^ Hourly observations of ambient temperature, relative humidity, solar radiation, wind speed, precipitation, and air pressure were extracted. Air pollutant concentrations, hourly observations of particulate pollutants (fine particulate matter [PM_2.5_] and suspended particulate matter), and gaseous pollutants (photochemical oxidants, sulfur dioxide, nitrogen dioxide, and carbon monoxide [CO]) were retrieved from the National Institute for Environmental Studies database.^12^ There were approximately 1,900 air pollution monitoring stations distributed throughout Japan. The respective observations were based on measurements at ambient air quality monitoring stations, which are representative of the air quality in the area. For CO, measurements from roadside stations were included due to limited availability of nearby monitoring stations. If data were missing for more than three consecutive hours at the nearest station, the next nearest station’s data were used. If an observation was missing for up to 2 consecutive hours, the average value of the previous and following observations was used.

### Statistical analysis

Observed meteorological and air pollutant data were indexed to the marathon start time, and the value at the start time was used as the exposure for the main analysis. Continuous variables were assessed for approximate normality by visual inspection of histograms and Q-Q plots and by the Shapiro-Wilk test. Approximately normally distributed variables were summarized as mean (SD) and compared using t tests; non-normally distributed variables were summarized as median (Q1–Q3) and compared using Wilcoxon rank-sum tests. Categorical variables were compared using chi-square or Fisher exact tests, as appropriate. The main analysis was based on a generalized linear model with a Poisson distribution for the number of SCA cases in each race, with the logarithm of the number of race participants included as an offset. Given the low event rate and the absence of material evidence suggesting model misfit, the Poisson regression model was retained as the primary analytic framework. Results are presented as incidence rate ratios (IRRs) and their 95% CIs, calculated per 1-unit increase for all covariates except CO, which was calculated per 0.1 ppm increase. Univariable analyses were used to evaluate each environmental variable separately, whereas multivariable analyses evaluated 2 combinations of environmental and demographic variables. Model 1 adjusted for latitude, longitude, elevation above sea level, and marathon start time. Model 2 additionally adjusted for race-level participant composition, including the proportions of participants aged ≥60 years and the proportion of male participants, to account for potential differences in participant demographics across races. To further explore the shape of the association with temperature, which was found to be significantly associated with the outcome in the Poisson regression models, a restricted cubic spline model was fit. For the spline analysis, the relative risk was expressed in reference to the median temperature, with knots placed at the 25th and 75th percentiles of the temperature distribution. All statistical analyses were performed using R (version 4.4.1; R Core Team, 2024).

### Sensitivity analysis

We performed additional analyses to examine the robustness of the main analysis. First, wet bulb globe temperature (WBGT), a commonly used index for stratifying thermal health risk during road races, was estimated from the available meteorological data.^13^ Because WBGT was not directly measured at each race, values were calculated using the estimation formula proposed by Ono et al^14^ in the Supplemental Methods. Second, the outcome was restricted to defibrillated cases, defined as cases in which a shockable rhythm was identified and a shock was delivered. Third, the exposure metric was changed from the start-time value to the average value over the 3-hour period spanning 1 hour before to 1 hour after the race start. Fourth, women-only races and races in which the 25-mile split time for the final runner was <4 hours were excluded to reduce heterogeneity in participant characteristics across races. Fifth, races in which the distance between the starting point and the monitoring station exceeded 13.1 miles were excluded to reduce potential exposure misclassification. Sixth, analyses were repeated after excluding races held in highly urbanized cities, defined as host cities with a population density exceeding 5,000 inhabitants per square km to reduce potential confounding by unmeasured urban environmental factors.

## Results

Between April 2011 and March 2020, 571 JAAF-certified full marathon races were held (Figure 1, Supplemental Figure 1), with a total of 4,528,134 runners involved. Data on the distribution of sex and age groups for each race were available for 95.8% and 80.6%, respectively. The proportion of male participants was 84.7%, and the age distribution was also broadly similar across races (Table 1).

**Table 1:** Characteristics of Marathon Races

Number of participants	6,258 (2028, 11,520)
Age group^a^, %	
<40 years	34.0 (29.2, 38.5)
40–59 years	56.5 (52.8, 60.8)
≥60 years	9.0 (6.8, 11)
Male^b^, %	84.7 (80.6, 88.0)
Completion rate^c^, %	89.9 (84.0, 93.3)
Season^d^, n (%)	
Autumn	218 (38.2)
Winter	208 (36.4)
Spring	131 (22.9)
Summer	14 (2.5)
Start time, hour:minute	9:00 (9:00, 10:00)
Latitude at starting site^e^, degree	35.0 (33.7, 36.4)
Longitude at starting site^e^, degree	135.9 (132.8, 139.6)
Elevation from sea level, m	12 (3, 52)

Values are median (Q1, Q3) unless otherwise indicated.

JAAF = Japan Association of Athletics Federations; Q1 = 1st quartile; Q3 = 3rd quartile.

a Age-group data were available for 460 of 571 races (81%).

b Sex-distribution data were available for 547 of 571 races (96%).

c The ratio of the number of finishers to the number of participants.

d Autumn was defined as September to November, winter as December to February, spring as March to May and summer as June to August.

e Numerical values are expressed in the decimal degrees.

Of the 4.53 million participants, 75 SCA cases occurred in 62 races (rate per 100,000: 1.66; 95% CI: 1.30-2.08) the median age of SCA patients was 52 years (Q1–Q3: 45-61), and 70 (93.3%) were males. Shockable rhythms were detected by automated external defibrillators, and defibrillation was delivered in 55 runners (73.3%). Return of spontaneous circulation at the race site was documented in 70 of the 75 cases (93.3%). Four additional patients achieved return of spontaneous circulation during transport or after hospital arrival, and only 1 death was reported (Supplemental Table 1). Among cases with available collapse-location data, SCA events occurred throughout the race course, but more than half (58.7%) occurred in the later segments, including near and after completion (Supplemental Figure 2).

A summary of the meteorological and air pollution data for each race is presented in Table 2. Marathons were held predominantly in autumn and winter, and the mean ambient temperature at race start was 11.5 °C (SD 5.8). The temperature exceeded 20 °C in only 39 races (6.8%). Air pollutant concentrations at race start were generally low. Races with SCA had numerically lower ambient temperatures (10.5 °C [SD 5.3] vs 11.7 °C [SD 5.8]; *P* = 0.12), and higher air pressure than races without SCA (1,015 hPa [SD 7.1] vs 1,011 hPa [SD 18], *P* < 0.001). Among air pollutants, nitrogen dioxide was higher in races with SCA, whereas other pollutants did not differ materially between groups. Among SCA cases with available collapse-time data, the temperature at collapse was modestly higher than the race-start temperature, with a median change of 2.2 °C (Q1–Q3: 1.2-4.3) (Supplemental Table 1). The median distance from the starting point to the nearest weather station measuring temperature was 3 km (Q1–Q3: 1.7-6.5), and that to the PM_2.5_ monitoring station was 5 km (Q1–Q3: 2.2-11.9).

**Table 2:** Meteorological and Air Pollution Data for Races With and Without Sudden Cardiac Arrest

	Overall (N = 571)	Non-SCA Race (N = 509)	SCA Race (N = 62)	*P* Value
Meteorological variables				
Temperature, °C	11.5 (5.8)	11.7 (5.8)	10.5 (5.3)	0.12
Humidity, %	66.3 (17.3)	66.5 (17.4)	65.1 (16.4)	0.52
Solar radiation, MJ/m^2^	1.3 [0.7, 1.8]	1.3 [0.7, 1.8]	1.3 [0.7, 1.7]	0.69
Wind speed, m/s	2.5 (1.8)	2.5 (1.9)	2.6 (1.7)	0.55
Precipitation, mm/h	0 [0, 0]	0 [0, 0]	0 [0, 0]	0.48
Air pressure, hPa	1,011 (17)	1,011 (18)	1,015 (7.1)	<0.001
Air pollution variables				
Fine particulate matter, μg/m^3^	10.3 [6.0, 17]	10.0 [6.0, 17]	10.0 [6.0, 16]	0.53
Suspended particulate matter, μg/m^3^	14 [7.0, 22]	14 [7.8, 22]	14 [6.5, 21]	0.45
Photochemical oxidants, ppb	27 [16, 36]	28 [16, 36]	25 [15, 34]	0.41
Sulfur dioxide, ppb	1.0 [1.0, 3.0]	1.0 [0.0, 2.2]	1.0 [1.0, 3.0]	0.33
Nitrogen dioxide, ppb	6.0 [3.3, 10]	6.0 [3.0, 10]	8.0 [5.0, 15]	0.002
Carbon monoxide, 0.1 ppm	4.0 [3.0, 5.0]	4.0 [3.0, 5.0]	4.0 [3.0, 5.0]	0.068

MJ = megajoule; hPa = hectopascal; ppb = parts per billion; ppm = parts per million; SCA = sudden cardiac arrest.

Table 3 shows the results of the Poisson regression analyses for meteorological and air pollutant variables in relation to SCA. No variable was significantly associated with SCA in the univariable analyses. In the multivariable analyses, race-start temperature was inversely associated with SCA risk, with IRRs per 1 °C increase of 0.95 (95% CI: 0.91-0.99) in model 1 and 0.94 (95% CI: 0.90-0.99) in model 2. These findings are summarized in the Central Illustration. Restricted cubic spline analysis also supported an inverse association, with the risk of SCA tending to increase at lower temperatures, although the CI widened at the extremes of the temperature distribution (Supplemental Figure 3).

**Table 3:** Associations of Each Meteorological and Pollutant Measurement With Sudden Cardiac Arrest During Marathons

	Univariable		Multivariable			
	IRR	95% CI	Model 1		Model 2	
			IRR	95% CI	IRR	95% CI
Temperature, per 1 °C increase	0.969	0.932-1.007	0.954	0.912-0.997	0.942	0.895-0.991
Humidity, per 1% increase	0.999	0.985-1.012	1.000	0.986-1.014	0.996	0.981-1.011
Solar radiation, per 1 MJ/m^2^ increase	0.914	0.662-1.263	0.894	0.637-1.255	0.946	0.652-1.374
Wind speed, per 1 m/s increase	0.983	0.865-1.116	0.958	0.837-1.096	0.977	0.838-1.139
Precipitation, per 1 mm/h increase	1.033	0.810-1.319	1.018	0.798-1.298	0.372	0.079-1.756
Air pressure, per 1 hPa increase	1.020	0.997-1.044	1.017	0.987-1.048	1.012	0.977-1.049
Fine particulate matter, per 1 μg/m^3^ increase	0.986	0.959-1.013	0.984	0.957-1.012	0.984	0.955-1.015
Suspended particulate matter, per 1 μg/m^3^ increase	0.988	0.967-1.009	0.984	0.962-1.006	0.976	0.952-1.001
Photochemical oxidants, per 1 ppb increase	0.997	0.979-1.016	0.993	0.974-1.013	0.999	0.977-1.021
Sulfur dioxide, per 1 ppb increase	1.020	0.907-1.148	1.026	0.908-1.159	1.014	0.887-1.158
Nitrogen dioxide, per 1 ppb increase	1.002	0.973-1.032	0.999	0.969-1.030	0.991	0.958-1.026
Carbon monoxide, per 0.1 ppm increase	1.025	0.930-1.130	1.018	0.917-1.129	1.005	0.877-1.152

IRR = incidence rate ratio; other abbreviations as in Table 2.

Model 1 adjusted for latitude, longitude, elevation above sea level, and marathon start time. Model 2 adjusted for latitude, longitude, elevation above sea level, marathon start time, the proportion of participants aged ≥60 years, and the proportion of male participants.

**Central Illustration:**
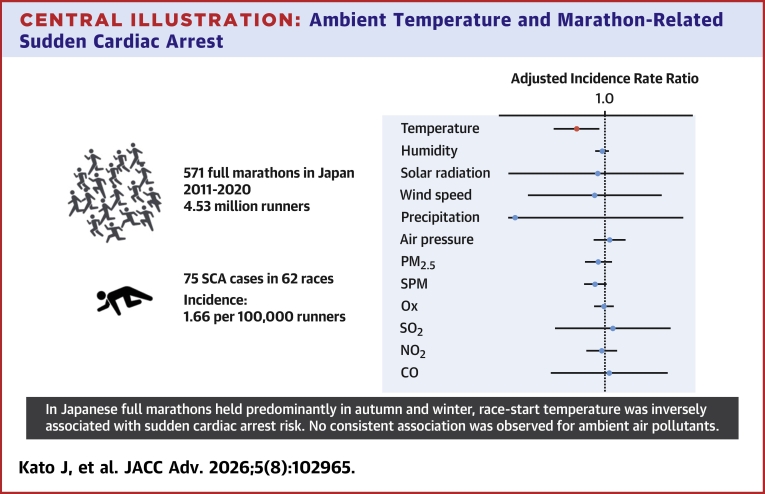
Ambient Temperature and Marathon-Related Sudden Cardiac Arrest This illustration summarizes the study population and adjusted incidence rate ratios from model 2 for sudden cardiac arrest according to meteorological and air pollution variables at race start in Japanese marathons. Model 2 adjusted for latitude, longitude, elevation above sea level, marathon start time, the proportion of participants aged ≥60 years, and the proportion of male participants. Incidence rate ratios represent the change in sudden cardiac arrest risk per 1-unit increase in each variable, except for carbon monoxide, for which the estimate is shown per 0.1 ppm increase. For temperature, an incidence rate ratio below 1.0 corresponds to higher SCA risk at lower race-start temperatures. CO = carbon monoxide; NO_2_ = nitrogen dioxide; Ox = photochemical oxidants; PM_2.5_ = fine particulate matter; SCA = sudden cardiac arrest; SO_2_ = sulfur dioxide; SPM = suspended particulate matter.

Several sensitivity analyses showed results broadly similar to those of the main analysis. First, when WBGT was used instead of ambient temperature, the direction and magnitude of the association remained similar in the multivariable analyses (model 1: IRR: 0.96; 95% CI: 0.91-1.01; model 2: IRR: 0.95; 95% CI: 0.90-1.00). Analyses restricting the outcome to defibrillated cases (Supplemental Table 2), redefining exposure as the 3-hour average from 1 hour before to 1 hour after the race start (Supplemental Table 3), excluding women-only and potentially elite races (Supplemental Table 4), excluding races with distant monitoring stations (Supplemental Table 5), and excluding races held in cities with high population density (Supplemental Table 6) all produced similar overall findings, with the inverse association between lower ambient temperature and SCA generally preserved. No consistent association was observed for any air pollutant across the main and sensitivity analyses.

## Discussion

### Main findings

In this cohort of marathon races held predominantly in autumn and winter, lower ambient temperature at marathon race start was significantly associated with a higher risk of SCA, with each 1 °C decrease corresponding to an approximately 5% to 6% increase in risk. No significant associations were observed for other meteorological variables or air pollutants.

The overall incidence of SCA in our cohort was 1.66 per 100,000 runners, which was somewhat higher than that reported in the RACER 2 study. However, prior analyses of Japanese marathon runners have shown that SCA incidence increases sharply with age from 0.9 cases per 100,000 runners among those aged <40 years and those in their 40s to 2.6 among those in their 50s and 5.5 among those aged ≥60 years.^9^ These findings suggest that crude incidence is strongly influenced by the age composition of participants. Accordingly, differences in the proportion of older runners, particularly older men, may partly explain variations in overall incidence across registries.

### Association between ambient temperature and sudden cardiac arrest

This study provides evidence of an association between lower ambient temperature and SCA during marathon races. This finding is consistent with epidemiological evidence from the general population, in which colder temperatures have been associated with an increased incidence of acute myocardial infarction,^15^ out-of-hospital cardiac arrest,^16^ and ventricular arrhythmias.^17^ Together, these observations suggest that cold exposure may act as a trigger for acute cardiovascular events, even in physically active populations.

Exposure to cold environments may increase several physiological responses aimed at preserving core body temperature, including peripheral vasoconstriction mediated by sympathetic activation and heat production through shivering. These responses may be accompanied by increases in blood pressure and heart rate, thereby increasing cardiac workload.^18^ In addition, cold exposure has also been associated with electrophysiological changes, including QT prolongation,^19^ and hemorheological changes that may further increase vulnerability to acute cardiovascular events.^20^

The cardiovascular response to endurance exercise under cold conditions is likely to be multifactorial. During marathon running, the combination of cold-induced vasoconstriction and high metabolic demand may increase hemodynamic stress and reduce cardiovascular reserve. In susceptible individuals, these responses could plausibly promote myocardial ischemia, ventricular arrhythmia, or both. Previous cohort studies have shown that coronary artery disease accounts for a substantial proportion of SCA cases during long-distance road races.^1^, ^2^, ^3^ However, the present study did not ascertain hospital diagnoses or the specific etiology of each event; therefore, these mechanisms should be interpreted as hypothesis-generating rather than causal evidence.

### Association of air pollutants with sudden cardiac arrest

In the present study, we did not observe a statistically significant association between ambient air pollutants and SCA during marathons. Previous studies, however, have shown that higher concentrations of particulate and gaseous air pollutants are associated with an increased risk of acute myocardial infarction^21^^,^^22^ and out-of-hospital cardiac arrest^23^^,^^24^ in the general population. Air quality also remains relevant to endurance events, as pollutants such as particulate matter have been associated with impaired marathon performance.^25^ In addition, Gerardin et al^26^ reported a higher incidence of cardiac events during road races conducted under elevated air pollution conditions.

Several factors may explain the absence of a clear association in our study. First, air pollutant concentrations during the included races were generally low. Second, our exposure assessment was based on area-level measurements around the race start rather than on each runner’s cumulative or repeated prior exposure. Because many participants were drawn from geographically diverse areas, race-day ambient concentrations may not have adequately captured the longer-term exposure history that could influence cardiovascular vulnerability. Third, even during the race itself, fixed-site monitoring values may have incompletely reflected each runner’s true inhaled exposure along the course. These sources of exposure misclassification may have attenuated any true association between air pollution and SCA. Further studies incorporating a wider range of pollution levels and more refined individual-level exposure assessment are warranted.

### Clinical relevance and future perspectives

Cold-weather marathons present both advantages and disadvantages, as cooler temperatures can enhance marathon performance^27^ while being associated with an increased risk of SCA. Because the included marathons were held predominantly in autumn and winter, this finding should be interpreted within the temperature range represented in this cohort and should not be taken as evidence against potential risk at higher temperature extremes. At the other end of the temperature spectrum, the increasing number of marathons held under hot conditions has raised separate concerns because of the risk of heat-related illness.^28^^,^^29^ Race medical preparedness should therefore consider temperature extremes in both directions, because warmer conditions remain relevant to heat-related illness, dehydration, electrolyte abnormalities, and other race-day medical emergencies. However, because only a small proportion of races in the present cohort were held under high-temperature conditions, further studies are needed to investigate whether higher temperature extremes are associated with SCA risk.

In practice, adjusting marathon dates or start times solely to reduce SCA risk is unlikely to be feasible. Rather, our findings may help race organizers, medical personnel, volunteers, and other stakeholders optimize preparedness. A shared awareness that colder temperatures are associated with an increased risk of SCA may support more effective planning of on-site medical response and emergency resource allocation, thereby improving safety for all participants.

### Study Limitations

This study has several limitations. First, the analysis was restricted to marathon races held in Japan, and the participants were almost exclusively Japanese. Therefore, the generalizability of our findings to other populations may be limited, although previous studies have reported broadly similar incidences of SCA during road races in both Eastern and Western countries.^1^, ^2^, ^3^, ^4^ Second, ambient air pollutant concentrations among the included races were generally low, which may partly explain the absence of statistically significant associations between air pollution and SCA risk. However, the observed levels, particularly for PM_2_._5_, were not markedly different from those reported for major marathon races in the United States.^30^ Third, some SCA cases may have been missed, particularly events occurring within 1 hour after the finish but outside the race venue or in private settings. Fourth, only a small proportion of races were conducted under high-temperature conditions, resulting in limited precision for estimating SCA risk at higher temperature ranges. Accordingly, the present findings do not exclude a potential increase in SCA risk at higher temperature extremes. Finally, individual-level information on underlying cardiovascular disease, cardiomyopathy, smoking status, body weight, physical fitness, socioeconomic status, ethnicity, other environmental exposures, and body temperature during the race was not available. In addition, hospital diagnoses and the specific etiology of each SCA event were not ascertained; therefore, noncoronary causes, including electrolyte abnormalities or stimulant use, could not be excluded. Although the main models accounted for race-level age and sex composition, residual confounding by unmeasured individual-level factors cannot be excluded.

## Conclusions

The incidence of SCA during marathon races in Japan was 1.66 per 100,000 runners. In Japanese marathon races held predominantly in autumn and winter, ambient temperature at race start was inversely associated with SCA risk. In contrast, no statistically significant association was observed between ambient air pollutants and SCA risk.

### Declaration of Generative AI and AI-Assisted Technologies in the Writing Process

During the preparation of this work, the first author used DeepL Pro and ChatGPT for language editing and drafting support. The authors reviewed and edited the content as needed and take full responsibility for the content of the manuscript.

Perspectives **COMPETENCY IN MEDICAL KNOWLEDGE:** In Japanese marathon races held predominantly in autumn and winter, lower ambient temperature at race start was associated with a higher risk of sudden cardiac arrest, whereas no significant association was observed for ambient air pollutants.

**TRANSLATIONAL OUTLOOK:** Further studies incorporating individual-level clinical data and more refined exposure assessment are needed to clarify the mechanisms linking cold exposure to sudden cardiac arrest during endurance events and to inform risk-adapted medical preparedness.

## Funding support and author disclosures

The authors have reported that they have no relationships relevant to the contents of this paper to disclose.
