# Supplementary material for: Meteorological and Environmental Factors Associated With Sudden Cardiac Arrest During Marathons in Japan
Source: JACC Adv. 2026 Jun 29;5(8):102965. doi: 10.1016/j.jacadv.2026.102965 (PMC13330661; doi:10.1016/j.jacadv.2026.102965)
Supplement: Supplemental Material [file mmc1.pdf]

INDEX

Supplementary Methods. Estimation of wet bulb globe temperatures (WBGT)........ 2
Supplementary Table 1. List of cases of sudden cardiac arrest during marathons........ 3
Supplementary Table 2. Associations of each meteorological and pollutant measurement with the provision of defibrillation by automated external defibrillator........ 11
Supplementary Table 3. Associations of meteorological and air pollutant variables averaged over the period from 1 hour before to 1 hour after marathon start with sudden cardiac arrest........ 12
Supplementary Table 4. Associations of each meteorological and pollutant measurement with cardiac arrest during marathons, excluding elite and women-only races (N = 508)........ 13
Supplementary Table 5. Associations between measurements of each meteorological and pollutant variable and cardiac arrest during marathons, excluding races where the measuring station is more than 13.1 miles from the starting site (N = 482)........ 14
Supplementary Table 6. Associations between measurements of each meteorological and pollutant variable and cardiac arrest during marathons, excluding host cities with more than 5,000 inhabitants / km^2 (N = 518)........ 15
Supplementary Figure 1. Flow chart of the study........ 16
Supplementary Figure 2. Distribution of sudden cardiac arrest events........ 17
Supplementary Figure 3. Non-linear relationship between sudden cardiac arrest and ambient temperature........ 18

Supplementary Methods. Estimation of wet bulb globe temperatures (WBGT).

The following formula was created based on the WBGT values measured at six meteorological stations in Japan from 2007 to 2009. Using the measured values for 2008 as a reference, the error margin for the estimated values within ±1°C, ±0.5°C, and ±0.1°C was 97.1–99.6%, 81.5–91%, and 27.1–33.2%, respectively.

$$\mathrm{WBGT}\ (°\mathrm{C}) = 0.735 \times T_a + 0.0374 \times RH + 0.00292 \times T_a \times RH + 7.619 \times SR - 4.557 \times SR^2 - 0.0572 \times WS - 4.064$$

where T_a is air temperature (°C), *RH* is relative humidity (%), *SR* is total solar radiation (kW/m^2), and *WS* is mean wind speed (m/s).

Reference

Ono M, Tonouchi M. Estimation of wet-bulb globe temperature using generally measured meteorological indices (in Japanese). Jpn J Biometeor. 2014;50:147-157.

Supplementary Table 1. List of cases of sudden cardiac arrest during marathons

No.	Case of SCA (Age/Sex)	Time of event (month year)	Time from start to collapse (hour:min)	Distance from start to the site of collapse (km)	Race-start temperature (°C)	Temperature at collapse (°C)	Change from race-start temperature (°C)	First aid and the progress
1	30s/M	Dec 2011	2:20	15	19.3	21.4	2.1	CPR and defibrillation with an AED achieved ROSC on site.
2	30s/M	Jan 2012	2:50	28	2.3	5.0	2.7	Only CPR achieved ROSC on site.
3	60s/M	Jan 2012	3:10	25	7.7	10.9	3.2	Three minutes after the start of CPR, spontaneous circulation resumed on site after two defibrillations with an AED.
4	50s/M	Feb 2012	2:30	19	5.5	6.3	0.8	CPR and defibrillation with an AED achieved ROSC on site.
5	40s/M	May 2012	3:00	23	3.6	8.8	5.2	CPR and defibrillation with an AED achieved ROSC on site.
6	40s/M	May 2012	2:55	29	6.9	6.4	-0.5	CPR and defibrillation with an AED achieved ROSC on site.
7	60s/M	Nov 2012	4:15	42	7.7	13.9	6.2	CPR and two defibrillations with an AED achieved ROSC on site.
8	60s/M	Nov 2012	4:40	34	7.7	13.9	6.2	CPR and defibrillation with an AED achieved ROSC on site.

9	50s/M	Dec 2012	2:00	19	19.9	21.4	1.5	CPR and AED defibrillation failed to restore spontaneous circulation on site, and the patient regained consciousness after transfer to hospital.
10	30s/M	Feb 2013	2:40	23	5.0	7.1	2.1	CPR and defibrillation with an AED achieved ROSC on site.
11	60s/M	Feb 2013	3:30	21	5.0	6.8	1.8	CPR and defibrillation with an AED achieved ROSC on site.
12	50s/M	Aug 2013	3:50	40	23.9	21.1	-2.8	After three defibrillations with an AED, the rhythm became non-shockable and the patient was transported to hospital while continuing CPR. Spontaneous circulation was subsequently restored.
13	40s/M	Nov 2013	0:30	4	16.4	16.4	0	CPR and defibrillation with an AED achieved ROSC on site.
14	60s/M	Dec 2013	4:20	35	8.3	14.5	6.2	Spontaneous circulation resumed on site with CPR alone.
15	50s/M	Jan 2014	1:30	10	8.7	9.9	1.2	CPR and defibrillation with an AED achieved ROSC on site.
16	40s/M	Jan 2014	4:20	42	12.3	10.9	-1.4	CPR and defibrillation with an AED achieved ROSC on site.
17	50s/M	Feb 2014	2:20	18	5.9	8.8	2.9	Three defibrillations were administered after seven minutes of CPR, but death was confirmed after transport to hospital.

18	60s/M	May 2014	3:30	42	7.3	8.9	1.6	CPR and defibrillation with an AED were performed on site and spontaneous circulation resumed after transport to hospital.
19	60s/M	Apr 2014	4:10	42	9.7	12.5	2.8	CPR and defibrillation with an AED achieved ROSC on site.
20	30s/M	Nov 2014	3:20	41	13.9	18.3	4.4	CPR and two defibrillations with an AED achieved ROSC on site.
21	60s/M	Nov 2014	3:50	34	8.3	16.2	7.9	CPR and defibrillation with an AED achieved ROSC on site.
22	50s/M	Nov 2014	5:20	37	18.7	20.7	2	CPR and three defibrillations with an AED achieved ROSC on site.
23	50s/M	Dec 2014	0:20	1.5	6.5	6.5	0	CPR and defibrillation with an AED achieved ROSC on site.
24	30s/M	Feb 2015	0:30	3	13.5	13.5	0	Spontaneous circulation resumed on site with CPR alone.
25	50s/M	Feb 2015	3:30	38	8.1	6.3	-1.8	Spontaneous circulation resumed on site with CPR alone.
26	50s/M	May 2015	2:50	26	4.8	6.7	1.9	CPR and three defibrillations with an AED achieved ROSC on site.
27	40s/M	May 2015	4:05	41	4.8	7.3	2.5	CPR and defibrillation with an AED achieved ROSC on site.
28	70s/M	Oct 2015	0:15	0.7	17.2	17.2	0	CPR and defibrillation with an AED achieved ROSC on site.

29	30s/M	Oct 2015	2:15	33	17.2	19.2	2	CPR and defibrillation with an AED achieved ROSC on site.
30	50s/M	Nov 2015	5:15	Immediately after finish	12.4	14.7	2.7	CPR and defibrillation with an AED achieved ROSC on site.
31	70s/M	Nov 2015	0:55	7	13.7	16.7	3	Spontaneous circulation was resumed during ambulance transport while continuing CPR.
32	50s/M	Nov 2015	2:20	21	17.7	19.0	1.3	CPR and defibrillation with an AED achieved ROSC on site.
33	60s/M	Jan 2016	2:50	30	4.5	8.8	4.3	CPR and defibrillation with an AED achieved ROSC on site.
34	30s/M	Jan 2016	4:30	Immediately after finish	4.5	9.2	4.7	CPR and defibrillation with an AED achieved ROSC on site.
35	60s/M	Feb 2016	1:35	13	17.6	19.1	1.5	CPR and defibrillation with an AED achieved ROSC on site.
36	60s/M	Feb 2016	2:30	30	17.6	19.8	2.2	CPR and defibrillation with an AED achieved ROSC on site.
37	40s/M	Feb 2016	2:55	31	8.6	9.7	1.1	Spontaneous circulation resumed on site with CPR alone.
38	60s/M	Mar 2016	5:20	Immediately after finish	18.3	19.1	1.5	CPR and defibrillation with an AED achieved ROSC on site.
39	40s/M	Apr 2016	3:15	36	17.6	19.0	1.4	Spontaneous circulation resumed on site with CPR alone.

40	20s/M	Feb 2017	2:10	22	5.8	8.4	2.6	CPR and defibrillation with an AED achieved ROSC on site.
41	40s/M	Feb 2017	3:35	35	7.4	10.4	3.0	Spontaneous circulation resumed on site with CPR alone.
42	60s/M	Feb 2017	2:30	34	6.1	9.2	3.1	Spontaneous circulation resumed on site with CPR alone.
43	70s/M	Feb 2017	2:50	No record	16.4	18.6	2.2	CPR and defibrillation with an AED achieved ROSC on site.
44	30s/M	Feb 2017	3:25	37	8.6	13.9	5.3	CPR and defibrillation with an AED achieved ROSC on site.
45	30s/F	Mar 2017	4:25	Immediately after finish	7.5	13.0	3.7	CPR and defibrillation with an AED achieved ROSC on site.
46	20s/F	Mar 2017	5:05	41	6.3	13.8	7.5	Spontaneous circulation resumed on site with CPR alone.
47	40s/M	Mar 2017	3:55	Immediately after finish	6.3	14	7.7	Spontaneous circulation resumed on site with CPR alone.
48	40s/M	Oct 2017	2:30	25	17.6	19.3	1.7	CPR and defibrillation with an AED achieved ROSC on site.
49	50s/M	Nov 2017	2:35	24	9.3	13.0	3.7	CPR and defibrillation with an AED achieved ROSC on site.
50	60s/M	Nov 2017	4:20	42	9.3	14.4	5.1	CPR and defibrillation with an AED achieved ROSC on site.

51	40s/M	Nov 2017	4:20	32	11.8	10.2	-1.6	CPR and defibrillation with an AED achieved ROSC on site.
52	20s/F	Nov 2017	2:00	23	7.1	10.1	3	Spontaneous circulation resumed on site with 3 min of CPR.
53	30s/M	Dec 2017	0:25	7	4.0	4.0	0	Spontaneous circulation resumed on site with CPR alone.
54	50s/M	Dec 2017	1:45	15	4.0	6.3	2.3	CPR and defibrillation with an AED achieved ROSC on site.
55	20s/M	Feb 2018	3:50	42	4.7	5.6	0.9	CPR and defibrillation with an AED achieved ROSC on site.
56	50s/M	Feb 2018	2:30	23	4.7	8.4	3.7	Spontaneous circulation resumed on site with CPR alone.
57	50s/M	Feb 2018	2:20	19	6.0	7.2	1.2	CPR and defibrillation with an AED achieved ROSC on site.
58	50s/M	Feb 2018	3:30	37	6.0	7.1	1.1	CPR and defibrillation with an AED achieved ROSC on site.
59	50s/M	Feb 2018	4:40	30	6.0	7.4	1.4	CPR and defibrillation with an AED achieved ROSC on site.
60	40s/M	Mar 2018	No record	24	5.2	No record		Spontaneous circulation resumed on site with CPR alone.
61	50s/M	Mar 2018	5:20	Immediately after finish	9.5	15.0	5.5	Spontaneous circulation resumed on site with CPR alone.

62	40s/M	Mar 2018	3:20	35	10.5	16.3	5.8	CPR and defibrillation with an AED achieved ROSC on site.
63	60s/M	Mar 2018	5:30	50 min after finish	15.3	21.3	6.0	Spontaneous circulation resumed on site with CPR alone.
64	50s/M	Oct 2018	2:40	16	13.5	15.3	1.8	CPR and defibrillation with an AED achieved ROSC on site.
65	50s/M	Nov 2018	3:10	34	9	17.2	8.2	CPR and defibrillation with an AED achieved ROSC on site.
66	40s/M	Nov 2018	0:40	2	9.3	9.3	0	CPR and defibrillation with an AED achieved ROSC on site.
67	60s/M	Dec 2018	0:30	6	6.5	6.5	0	CPR and 2 defibrillations with an AED achieved ROSC on site.
68	40s/M	Feb 2019	3:20	Immediately after finish	6.7	11.2	4.5	Spontaneous circulation resumed on site with CPR alone.
69	50s/M	Feb 2019	4:20	37	6.7	11.5	4.8	CPR and 4 defibrillations with an AED achieved ROSC on site.
70	50s/M	Mar 2019	4:30	42	13.9	16.9	3	CPR and defibrillation with an AED achieved ROSC on site.
71	60s/M	Nov 2019	5:10	40 min after finish	17.2	18.3	1.1	Spontaneous circulation resumed on site with CPR alone.
72	40s/F	Dec 2019	No record	6	23.5	No record		CPR and defibrillation with an AED achieved ROSC on site.

73	50s/M	Dec 2019	3:15	29	7.5	13.7	6.2	CPR and defibrillation with an AED achieved ROSC on site.
74	40s/M	Feb 2020	4:30	Immediately after finish	6.4	8.0	1.6	CPR and defibrillation with an AED achieved ROSC on site.
75	40s/F	Feb 2020	3:50	40	11.1	13.4	2.3	CPR and defibrillation with an AED achieved ROSC on site.

SCA, sudden cardiac arrest; CPR, cardiopulmonary resuscitation; AED, automated external defibrillator; ROSC, return of spontaneous circulation.

Supplementary Table 2. Associations of each meteorological and pollutant measurement with the provision of defibrillation by automated external defibrillator.

	Univariable		Multivariable			
			Model 1		Model 2	
	IRR	95% CI	IRR	95% CI	IRR	95% CI
**Temperature, per 1 °C increase**	0.982	0.939, 1.028	0.972	0.923, 1.022	0.956	0.901, 1.015
**Humidity, per 1 % increase**	1.005	0.989, 1.021	1.007	0.991, 1.024	1.005	0.987, 1.022
**Solar radiation, per 1 MJ/m^2 increase**	0.830	0.566, 1.217	0.803	0.538, 1.197	0.809	0.522, 1.254
**Wind speed, per 1 m/s increase**	0.995	0.859, 1.153	0.973	0.832, 1.137	1.000	0.837, 1.195
**Precipitation, per 1 mm/h increase**	1.102	0.877, 1.385	1.088	0.861, 1.374	0.496	0.120, 2.051
**Air pressure, per 1 hPa increase**	1.013	0.990, 1.036	1.005	0.970, 1.041	1.006	0.965, 1.049
**Fine particulate matter, per 1 $\mu g/m^3$ increase**	0.980	0.947, 1.013	0.978	0.945, 1.012	0.982	0.946, 1.019
**Suspended particulate matter, per 1 $\mu g/m^3$ increase**	0.984	0.958, 1.009	0.980	0.954, 1.007	0.975	0.946, 1.005
**Photochemical oxidants, per 1 ppb increase**	0.988	0.966, 1.010	0.984	0.962, 1.006	0.983	0.957, 1.009
**Sulfur dioxide, per 1 ppb increase**	1.037	0.906, 1.186	1.046	0.911, 1.201	1.042	0.895, 1.212
**Nitrogen dioxide, per 1 ppb increase**	1.002	0.967, 1.037	0.998	0.963, 1.035	0.994	0.954, 1.035
**Carbon monoxide, per 0.1 ppm increase**	1.020	0.908, 1.145	1.013	0.895, 1.146	1.026	0.873, 1.207

Supplementary Table 3. Associations of meteorological and air pollutant variables averaged over the period from 1 hour before to 1 hour after marathon start with sudden cardiac arrest.

	**Univariable**		**Multivariable**			
			Model 1		Model 2	
	IRR	95% CI	IRR	95% CI	IRR	95% CI
**Temperature, per 1 °C increase**	0.966	0.929, 1.005	0.951	0.909, 0.995	0.938	0.890, 0.988
**Humidity, per 1 % increase**	1.000	0.986, 1.014	1.002	0.987, 1.016	0.998	0.983, 1.013
**Solar radiation, per 1 MJ/m^2 increase**	0.937	0.662, 1.328	0.924	0.642, 1.330	0.980	0.657, 1.462
**Wind speed, per 1 m/s increase**	0.959	0.834, 1.103	0.928	0.799, 1.079	0.944	0.797, 1.119
**Precipitation, per 1 mm/h increase**	0.963	0.671, 1.383	0.953	0.668, 1.359	0.238	0.033, 1.703
**Air pressure, per 1 hPa increase**	1.020	0.997, 1.044	1.017	0.987, 1.048	1.012	0.977, 1.049
**Fine particulate matter, per 1 μg/m^3 increase**	0.986	0.958, 1.016	0.984	0.955, 1.014	0.982	0.950, 1.015
**Suspended particulate matter, per 1 μg/m^3 increase**	0.988	0.966, 1.011	0.984	0.961, 1.008	0.980	0.954, 1.006
**Photochemical oxidants, per 1 ppb increase**	0.997	0.978, 1.017	0.993	0.973, 1.013	0.999	0.976, 1.022
**Sulfur dioxide, per 1 ppb increase**	1.030	0.911, 1.165	1.038	0.914, 1.180	1.030	0.894, 1.186
**Nitrogen dioxide, per 1 ppb increase**	1.001	0.969, 1.034	0.997	0.964, 1.031	0.989	0.952, 1.028
**Carbon monoxide, per 0.1 ppm increase**	1.017	0.914, 1.132	1.008	0.901, 1.128	0.999	0.863, 1.158

Supplementary Table 4. Associations of each meteorological and pollutant measurement with cardiac arrest during marathons, excluding elite and women-only races (N = 508).

	Univariable		Multivariable			
			Model 1		Model 2	
	IRR	95% CI	IRR	95% CI	IRR	95% CI
**Temperature, per 1 °C increase**	0.969	0.932, 1.007	0.957	0.915, 1.000	0.945	0.898, 0.994
**Humidity, per 1 % increase**	0.998	0.985, 1.012	1.000	0.986, 1.015	0.997	0.982, 1.012
**Solar radiation, per 1 MJ/m^2 increase**	0.914	0.656, 1.272	0.876	0.620, 1.238	0.926	0.637, 1.346
**Wind speed, per 1 m/s increase**	0.992	0.875, 1.126	0.969	0.847, 1.107	0.992	0.851, 1.157
**Precipitation, per 1 mm/h increase**	1.031	0.808, 1.316	1.014	0.795, 1.293	0.380	0.081, 1.776
**Air pressure, per 1 hPa increase**	1.019	0.997, 1.043	1.012	0.981, 1.044	1.009	0.973, 1.047
**Fine particulate matter, per 1 $\mu g/m^3$ increase**	0.985	0.957, 1.013	0.982	0.954, 1.011	0.980	0.950, 1.011
**Suspended particulate matter, per 1 $\mu g/m^3$ increase**	0.988	0.967, 1.010	0.984	0.961, 1.006	0.974	0.950, 1.000
**Photochemical oxidants, per 1 ppb increase**	0.997	0.978, 1.016	0.992	0.973, 1.011	0.997	0.976, 1.020
**Sulfur dioxide, per 1 ppb increase**	1.025	0.910, 1.155	1.027	0.908, 1.162	1.011	0.883, 1.158
**Nitrogen dioxide, per 1 ppb increase**	1.002	0.973, 1.033	1.001	0.971, 1.032	0.992	0.958, 1.027
**Carbon monoxide, per 0.1 ppm increase**	1.028	0.933, 1.133	1.023	0.922, 1.136	1.003	0.874, 1.151

Supplementary Table 5. Associations between measurements of each meteorological and pollutant variable and cardiac arrest during marathons, excluding races where the measuring station is more than 13.1 miles from the starting site (N = 482).

	**Univariable**		**Multivariable**			
			Model 1		Model 2	
	IRR	95% CI	IRR	95% CI	IRR	95% CI
**Temperature, per 1 °C increase**	0.968	0.929, 1.008	0.950	0.906, 0.996	0.943	0.896, 0.993
**Humidity, per 1 % increase**	0.999	0.986, 1.013	1.000	0.986, 1.015	0.997	0.981, 1.012
**Solar radiation, per 1 MJ/m^2 increase**	0.953	0.681, 1.333	0.937	0.659, 1.332	0.995	0.677, 1.462
**Wind speed, per 1 m/s increase**	0.965	0.843, 1.106	0.938	0.811, 1.084	0.978	0.836, 1.144
**Precipitation, per 1 mm/h increase**	0.817	0.430, 1.552	0.821	0.432, 1.560	0.408	0.088, 1.885
**Air pressure, per 1 hPa increase**	1.019	0.996, 1.043	1.017	0.985, 1.049	1.013	0.976, 1.050
**Fine particulate matter, per 1 µg/m^3 increase**	0.983	0.953, 1.014	0.982	0.951, 1.013	0.980	0.947, 1.014
**Suspended particulate matter, per 1 µg/m^3 increase**	0.988	0.966, 1.010	0.984	0.961, 1.007	0.978	0.953, 1.003
**Photochemical oxidants, per 1 ppb increase**	0.995	0.975, 1.014	0.989	0.969, 1.010	0.997	0.975, 1.020
**Sulfur dioxide, per 1 ppb increase**	1.026	0.904, 1.165	1.041	0.911, 1.189	1.013	0.878, 1.169
**Nitrogen dioxide, per 1 ppb increase**	1.004	0.973, 1.036	1.001	0.970, 1.033	0.990	0.956, 1.026
**Carbon monoxide, per 0.1 ppm increase**	1.029	0.920, 1.152	1.030	0.916, 1.157	1.014	0.870, 1.181

Supplementary Table 6. Associations between measurements of each meteorological and pollutant variable and cardiac arrest during marathons, excluding host cities with more than 5,000 inhabitants / km^2 (N = 518).

	**Univariable**		**Multivariable**			
			Model 1		Model 2	
	IRR	95% CI	IRR	95% CI	IRR	95% CI
**Temperature, per 1 °C increase**	0.978	0.937, 1.020	0.960	0.914, 1.009	0.945	0.893, 1.001
**Humidity, per 1 % increase**	1.004	0.988, 1.020	1.005	0.988, 1.022	1.001	0.983, 1.020
**Solar radiation, per 1 MJ/m^2 increase**	0.836	0.568, 1.232	0.842	0.560, 1.265	0.913	0.584, 1.427
**Wind speed, per 1 m/s increase**	1.004	0.873, 1.154	0.977	0.842, 1.133	0.992	0.833, 1.180
**Precipitation, per 1 mm/h increase**	1.045	0.815, 1.339	1.023	0.798, 1.311	0.142	0.006, 3.356
**Air pressure, per 1 hPa increase**	1.020	0.994, 1.046	1.019	0.984, 1.055	1.018	0.974, 1.064
**Fine particulate matter, per 1 µg/m^3 increase**	0.995	0.966, 1.024	0.992	0.963, 1.023	0.993	0.962, 1.026
**Suspended particulate matter, per 1 µg/m^3 increase**	0.995	0.974, 1.016	0.991	0.966, 1.015	0.979	0.952, 1.007
**Photochemical oxidants, per 1 ppb increase**	0.995	0.974, 1.016	0.992	0.970, 1.014	0.994	0.969, 1.020
**Sulfur dioxide, per 1 ppb increase**	0.999	0.863, 1.157	1.002	0.862, 1.164	1.002	0.853, 1.177
**Nitrogen dioxide, per 1 ppb increase**	1.005	0.971, 1.040	1.001	0.966, 1.037	0.993	0.952, 1.035
**Carbon monoxide, per 0.1 ppm increase**	1.044	0.944, 1.155	1.034	0.928, 1.153	1.044	0.908, 1.201

Supplementary Figure 1. Flow chart of the study.

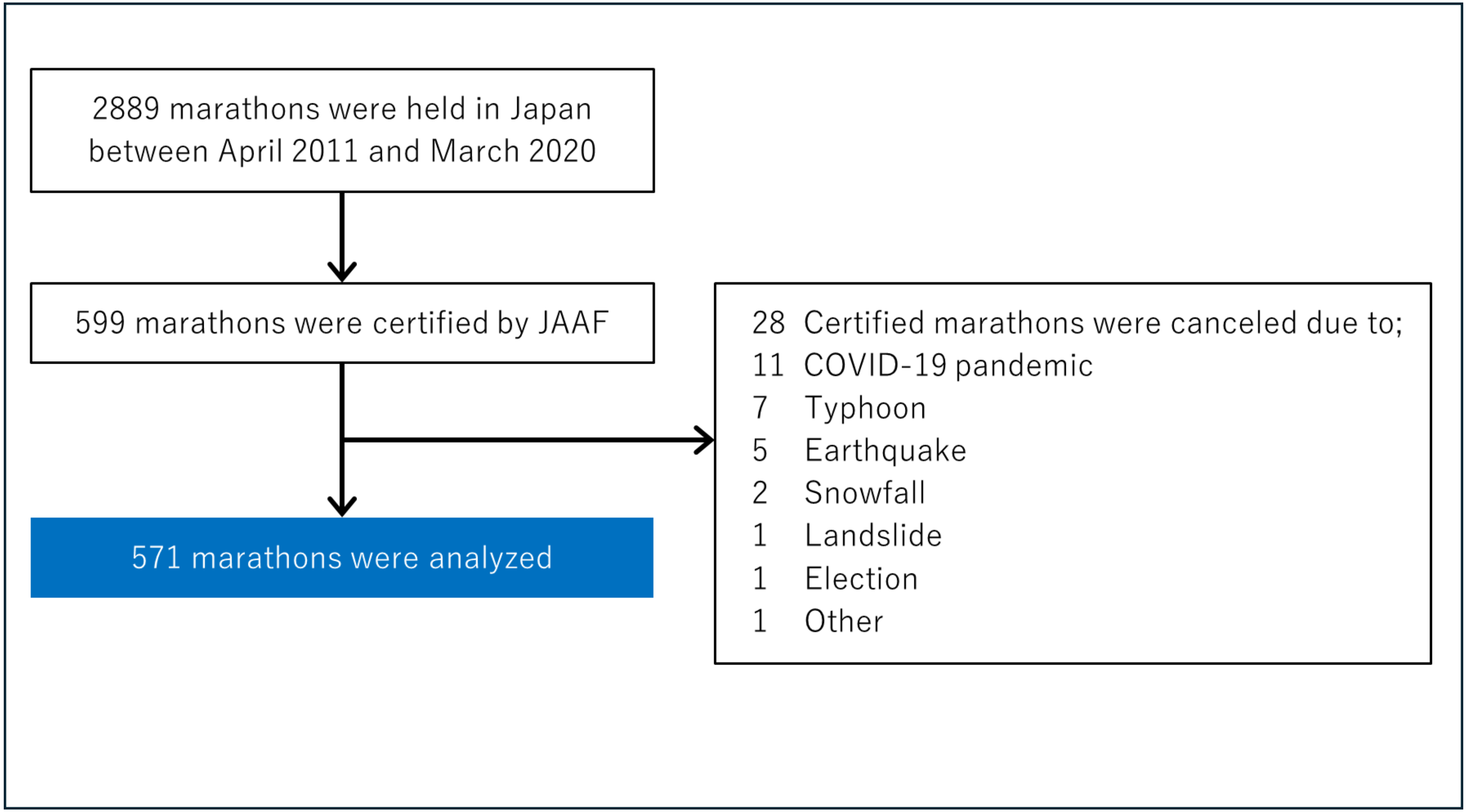

JAAF, Japan Association of Athletics Federations.

Supplementary Figure 2. Distribution of sudden cardiac arrest events

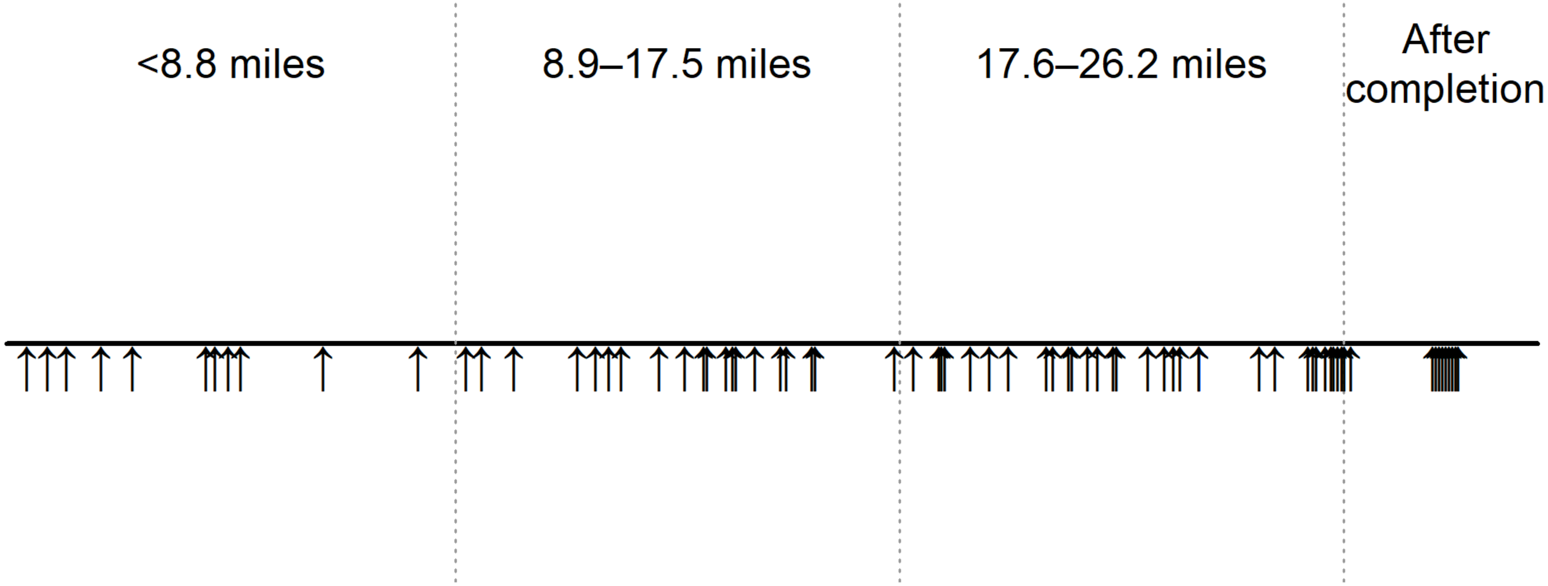

Arrows indicate the locations of sudden cardiac arrest events along the marathon course, expressed as distance from the start in miles. Vertical dotted lines divide the marathon course into three distance segments. Events occurring immediately after or after the finish are shown in the “After completion” category. To improve visualization, overlapping events were slightly offset horizontally.

Supplementary Figure 3. Non-linear relationship between sudden cardiac arrest and ambient temperature.

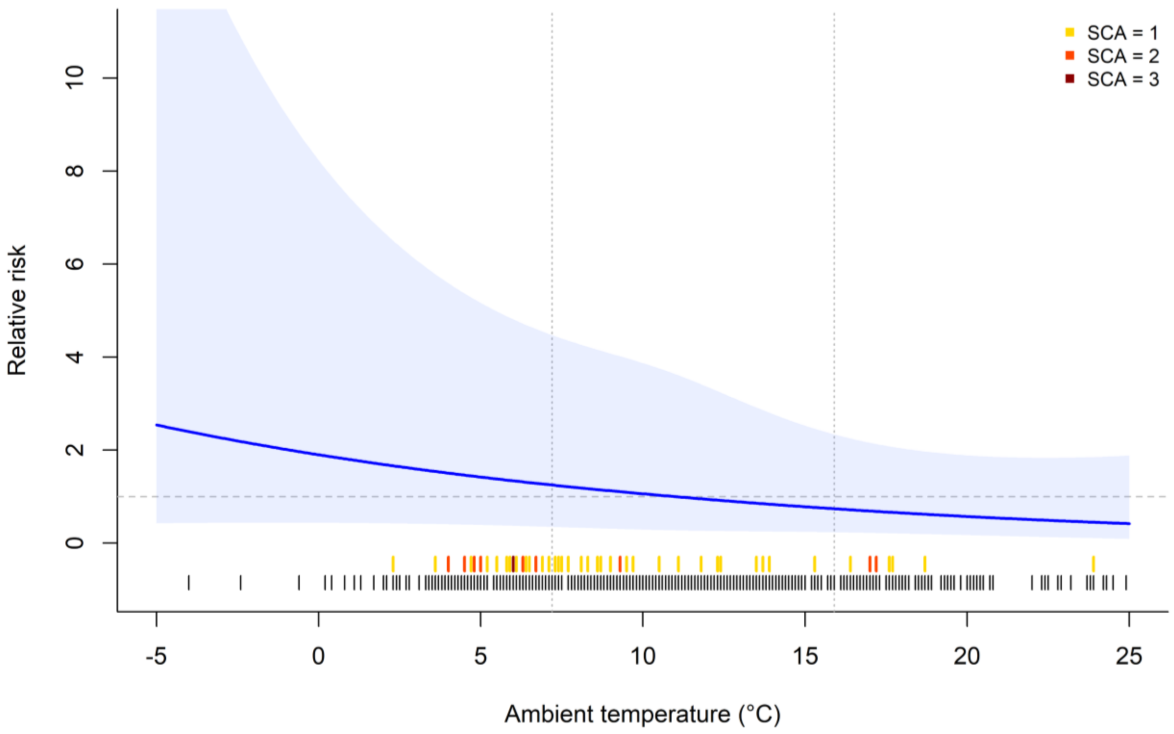

The blue curve represents the estimated relative risk of sudden cardiac arrest according to ambient temperature, derived from a Poisson regression model with restricted cubic splines. The model was adjusted for latitude, longitude, marathon start time, and elevation above sea level, with the logarithm of the number of participants included as an offset. Shaded areas indicate the 95% confidence intervals. Black vertical lines denote the distribution of individual marathon races. Colored vertical lines above indicate races in which sudden cardiac arrest occurred, with yellow representing one case, orange two cases, and dark red three cases. Risk estimates are expressed relative to the median temperature (11°C), with knots placed at the 25th and 75th percentiles of the temperature distribution. Confidence intervals were wide at the temperature extremes. SCA, sudden cardiac arrest.
